# Understanding Implementation Challenges to Genetic Testing for Familial Hypercholesterolemia in the United States

**DOI:** 10.3390/jpm9010009

**Published:** 2019-02-01

**Authors:** Rachele M. Hendricks-Sturrup, Christine Y. Lu

**Affiliations:** Precision Medicine Translational Research (PROMoTeR) Center, Department of Population Medicine, Harvard Pilgrim Health Care Institute and Harvard Medical School, Landmark Center, 401 Park Drive Suite 401, Boston, MA 02215, USA; christine_lu@harvardpilgrim.org

**Keywords:** implementation science, genetic testing, familial hypercholesterolemia, genomics, variants of unknown significance

## Abstract

Cardiovascular disease (CVD) is the leading cause of death in the United States (US), with familial hypercholesterolemia (FH) being a major inherited and genetic risk factor for premature CVD and atherosclerosis. Genetic testing has helped patients and providers confirm the presence of known pathogenic and likely pathogenic variations in FH-associated genes. Key organizations, such as the Centers for Disease Control and Prevention (CDC), American Heart Association (AHA), FH Foundation, and National Lipid Association (NLA), have recognized the clinical utility of FH genetic testing. However, FH genetic testing is underutilized in clinical practice in the US for reasons that are underexplored through the lens of implementation science. In this commentary, we discuss seven key implementation challenges that must be overcome to strengthen the clinical adoption of FH genetic testing in the US. These implementation challenges center on evidence of cost-effectiveness, navigating patient and provider preferences and concerns, gender and ethnic diversity and representation in genetic testing, and establishing clinical consensus around FH genetic testing based on the latest and most relevant research findings. Overcoming these implementation challenges is imperative to the mission of reducing CVD risk in the US.

## 1. Introduction

Cardiovascular disease (CVD) is a long-standing and leading cause of death in the United States (US) [1]. Familial hypercholesterolemia (FH) is a genetic cardiovascular condition associated with an increased risk of premature CVD and atherosclerosis. FH has an estimated prevalence of 1/250 person in the US, although prevalence varies by ethnicity. Clinically, FH is associated with high fasting or therapy-resistant low-density lipoprotein (LDL) cholesterol levels and a family history of premature CVD [2,3]. 

FH is inherited in a Mendelian and an autosomal dominant fashion. Genetic testing (e.g., multi-panel testing and genomic sequencing) can confirm variations in FH-associated genes/biomarkers in individuals. These genes/biomarkers are apolipoprotein B (ApoB), low density lipoprotein receptor (LDLR) and proprotein convertase subtilisin/kexin type 9 (PCSK9), lipase A (LIPA), signal-transducing adaptor protein 1 (STAP1), and apolipoprotein E (ApoE). Variations in LDLR are the most common, followed by variations in ApoB then PCSK9 (70-95% of FH cases) [2,4,5,6,7,8,9]. Autosomal recessive FH is associated with variations in the LDLR adaptor protein 1 (LDLRAP1) [10].

The clinical utility of FH genetic testing is generally recognized by key organizations such as the Centers for Disease Control and Prevention (CDC), the American Heart Association (AHA), the FH Foundation, and the National Lipid Association (NLA) [11,12,13,14]. For instance, FH genetic testing falls under the CDC’s Office of Public Health Genomics’ Tier 1 genomic applications because clinical practice guidelines based on systematic review support genomic/genetic testing for FH among individuals whose relatives are diagnosed with FH [11,12]. Moreover, an international panel of experts convened by the FH Foundation suggested that FH genetic testing become the standard of care for individuals diagnosed with definite or probable FH [15]. Despite expert consensus and organizational efforts, FH genetic testing is underutilized in clinical practice in the US for reasons that are underexplored through the lens of implementation science. 

In this commentary we discuss seven key implementation challenges that must be overcome to strengthen the clinical adoption of FH genetic testing in the US. These are, as discussed in order in the text: (1) Addressing the cost-effectiveness and affordability of genetic testing; (2) identifying and addressing patient and provider concerns about discriminatory insurance underwriting; (3) navigating patient and provider preferences and concerns about ordering and receiving FH genetic test results; (4) ensuring gender and ethnic diversity and representation across populations screened for FH; (5) updating and clarifying FH genetic test indications based on the presence of certain FH phenotypes in relevant clinical guidelines; (6) determining best practices for communicating results from the reclassification of FH variants of unknown significance (VUS) and variants with conflicting evidence of pathogenicity at the patient-provider level; and (7) establishing a clearer pathogenicity assessment of FH variants to more accurately assess CVD risk in FH populations with various phenotypes.

## 2. Key FH Genetic Test Implementation Challenges 

The affordability of genetic/genomic testing programs varies based on the geographic location of the program (e.g., developed versus developing countries, socialized versus non-socialized health care systems, health system resources available in rural versus urban settings, etc.). Thus, the first key challenge is determining the cost-effectiveness of population-based FH genetic/genomic testing programs in the US health care market. After reviewing recent literature, we found that only one study published between the years 2016–2018 measured the cost-effectiveness of implementing its FH genetic testing program [16]. Specifically, it was reported that for every 100 persons screened over a 10-year time period, there were net gains of 24.95 life-years and 29.07 quality-adjusted life-years and an incremental cost-effectiveness ratio of $4155 per years of life saved and $3565 per quality-adjusted life-years gained (in Australian currency) [16]. Similar cost-effectiveness analyses should be regularly performed over time to identify myriad circumstances under which FH genetic testing would be cost-effective in the US. 

Health care policies and insurance coverages for genetic testing vary considerably within the US and internationally [17]. Moreover, countries with limited access to or less broadly available financial coverage of genetic testing may question the costs and benefits of FH genetic testing, especially since insurance payers deny reimbursement for genetic testing when testing is deemed investigational versus medically necessary [18,19,20,21,22]. There is recent literature debating this very topic and issue [23]. A new study in Minnesota called Cascade Screening for Hypercholesterolemia (CASH) will partially address this issue by determining if genetic testing performed as part of cascade screening among study participants with pathogenic or likely pathogenic FH variants will have utility and cost-effectiveness [24]. The CASH study is one of the very few, if not the only, genetic test-driven FH cascade screening initiatives in the US that incorporate cost-effectiveness analyses into their research aims. 

In a study reported by Setia et al., the participants received free genetic testing, which Setia et al. explained may have influenced or led to the observed 100% genetic test uptake among the participants. Outside the research settings, free genetic testing is unlikely or uncommon in non-socialized health care systems like that of the US [19]. Insurance coverage denial or minimal insurance coverage of genetic testing would potentially burden patients with large out-of-pocket costs or surprise bills [17,20,25,26]. 

The second key challenge is identifying and addressing patient and provider concerns about discriminatory insurance underwriting, insurance claim denial for FH genetic testing, or unanticipated social stigmas that may accompany genetic testing [27]. An open-ended survey was conducted in Minnesota among 197 participants to identify the participants’ concerns about FH genetic testing [28]. Some of the concerns identified were that insurance costs would increase with FH diagnosis and that financial concerns would stop families from acting on genetic information [28]. These concerns may be especially true for statin monotherapy-intolerant individuals who may rely on genetic testing to confirm the presence of FH variations in PCSK9 (as a causal factor) to gain insurance coverage for needed yet costly specialty medications (e.g., PCSK9 inhibitors) [29]. 

The third key challenge is navigating patient and provider preferences and behaviors as barriers and facilitators to FH genetic testing. The American College of Cardiology reported that only 25% of primary care providers, 24% of cardiologists, and 15% of cardiovascular team members order genetic testing to diagnose FH [30]. Instead, the most common risk factors used for FH diagnosis or to differentiate FH from other causes of hypercholesterolemia were (in descending order based on level of usage): family history, LDL cholesterol levels, physical signs, medical history, and age [30]. Reasons for the observed FH genetic test underutilization were not explored or reported. Pang et al. also reported relatively low uptake (60.7%) of genetic testing among their pediatric study cohort, yet reported three main causes of the observed low uptake: (i) the parents wished for their children to independently decide to receive genetic testing once the children reached 18 years; (ii) genetic testing was not consensual among both parents; and (iii) given its high specificity (96.6%), parents preferred cholesterol testing alone due to their concerns about the stigmatization of genetic testing [16]. Zimmerman et al. also identified two implementation barriers to FH genetic testing upon surveying a sample of primary care physicians in family medicine and internal medicine, and specialists in geriatrics, functional medicine, and pediatrics in Minnesota (*n* = 173, average of 23 years of experience) [31]. Implementation barriers found were genetic testing being out of the physicians’ scope of practice and lack of access to a genetics professional within a rural practice setting were reported [31]. Similarly, in 2015 George, Kovak, and Cox similarly identified shortages of genetic specialists in rural areas as a barrier to cascade screening for FH [32]. It is important to consider that although FH genetic testing may be of interest to patients and providers, the impact of genetic testing could be minimal if patients are standardly prescribed statin medication without any other potentially necessary modifications to treatment or lipid surveillance. 

The fourth key challenge is ensuring gender and ethnic diversity in FH genetic testing programs. Gender and ethnic diversity are needed to better characterize the penetrance of FH variations among and across ethnic populations and the general US population. Khera et al. reported slightly less LDLR variation penetrance among their more ethnically diverse study cohort (LDLR-86%, ApoB-13%, and PCSK9-0.6%), as compared to most studies involving mainly Caucasian populations with relatively higher LDLR variation penetrance [33,34]. Kullo and Bailey acknowledged their ethnically skewed enrollment as a limitation in their study design [24]. It is still possible, however, to observe FH variant diversity within a single ethnic population, as genetic diversity exists both within and across ethnic populations. For instance, Wang et al. discovered an uncommon FH variant/polymorphism within their Chinese study population [35]. Therefore, ethnic and geographic population diversity are important for FH screening programs that rely on genetic testing to identify and assess the penetrance of known FH variants and to discover new FH variants. Cultural competency and/or contemplation about cultural sensitivities and social stigmas that have existed and continue to exist within US are also important practices to consider. 

The fifth key challenge is updating and clarifying FH genetic test indications based on FH phenotypes listed in relevant clinical guidelines. A recent review of 10 clinical guidelines by Migliara et al. outlined and summarized key indications for FH genetic testing (see Table 2 in Migliara et al.) [36]. The guidelines shows a consensus that FH genetic testing is not needed among the general population when the following characteristics are present: acute illness, statin use, and/or when FH diagnosis is unlikely based on phenotype [36]. 

In children, however, genetic testing is indicated if cholesterol concentrations are >230 mg/dL or are in the >95th percentile for age and gender [36]. Genetic testing is generally indicated in children with LDL cholesterol levels >150 mg/dL, in treated children with LDL cholesterol levels >300 mg/dL, and in untreated children with LDL cholesterol >500 mg/dL [36]. Minicocci et al. challenged these guideline parameters by determining that an LDL cholesterol level of ≥187.5 mg/dL is the optimal threshold to generally discriminate genotype+ from genotype− children (*n* = 78) [37]. Pang et al. also challenged these guideline parameters when they found that a LDL cholesterol threshold of ≥63 mg/dL correctly classified 94.4% of genotype+ children from genotype- children with 92.8% sensitivity and 96.6% specificity (*n* = 148; detection rate of 56.8%) [16]. 

Clinical guidelines recommend genetic testing in adults with Dutch Lipid Clinical Network (DLCN) criteria scores >5. This recommendation was challenged by Séguro et al. who showed that, among a study cohort of individuals aged 44 ± 18 years, a DLCN score >8 is strongly correlated with the presence of a FH variant (*n* = 344) [36,38]. Abul-Husn et al. found that around 45% of genotype+ individuals in their study cohort received an unlikely FH diagnosis using DLCN criteria (*n* = 229) [34]. Amor-Salamanca et al. also found that 44% of heterozygous genotype+ adults in their study were not diagnosed with FH according to DLCN criteria [39]. Lastly, Wu et al. found that 79% of genotype+ parents of children with severe FH were not diagnosed with FH per DLCN criteria (*n* = 79). Thus, clinical guidelines should undergo timely revisions for updates based on a systematic review of relevant findings that challenge the cut-offs for when genetic testing could be indicated based on phenotypic data.

The sixth key challenge is determining best practices for communicating results from the reclassification of FH VUS and variants with conflicting evidence of pathogenicity at the patient-provider level. To date, there is an unclear pathogenicity assessment for nearly one-third of FH variants in the ClinVar database; of over 3000 variants listed, there are more than 500 VUS and more than 400 variants with conflicting interpretations of pathogenicity. Illustrating this issue are both Amor-Salamanca et al. and Séguro et al., who each discovered VUS among their study populations. Their discoveries suggest a possible need for perpetual reanalysis of VUS and reconfirmation of FH variant significance over time [38,39]. Tsai et al. and Garret et al. both explained that in the US, family studies for VUS are available at most commercial laboratories, although there are many access barriers that include onerous application processes and potential participation fees [40,41]. Moreover, guidelines for when and how to conduct genetic reanalysis/reclassification are currently lacking, which warrants the need for further research in this area. To assist patients with VUS, an initiative hosted by the University of Washington called ‘Find My Variant’ provides the public with a patient-driven VUS reclassification toolkit that can be sought and used independently by FH patients with VUSs. The toolkit can provide FH patients with education about their unique VUSs and help FH patients connect with their families, clinical laboratories, and/or research studies to learn more about their FH VUSs.

In further consideration of the fact that nearly one-third of FH variants in the ClinVar database have an unclear pathogenicity assessment, the seventh key challenge is establishing a clearer pathogenicity assessment of FH variants to more accurately assess CVD risk across various FH phenotypes. This is clinically important because studies show that either genetic testing or lipid screening alone do not properly identify all FH cases [34,37]. For instance, there are populations with an FH genotype but with either a delayed or no FH phenotype (non-penetrant FH), and populations with no known FH genotype but with one or more FH phenotype(s) [34,37]. Both groups would require long-term treatment, if needed, and/or a more tailored approach to lipid surveillance to help reduce premature CVD risk. Illustrating this issue are two recent studies that identified FH variants with unclear pathogenicity (Setia et al. and Vohnout et al.; LDLR variant c.2416_2417 insG and LDLR variant c.1414 G>T, respectively) upon proband identification and cascade screening [18,42]. Our search within the ClinVar database showed overall conflicting interpretations of the pathogenicity for both LDLR variants [43,44]. Therefore, functional studies are needed to further or better classify FH variants to allow for more accurate diagnosis and more precise clinical surveillance and screening to help mitigate CVD risk.

## 3. Conclusions

In summary, the implementation of FH genetic/genomic testing as part of FH screening is recommended by the CDC, AHA, FH Foundation, and NLA in the US for CVD prevention. FH is also a key topic of discussion among health scientists at institutions such as the National Academies of Science and Engineering, and the Medicine Board on Health Sciences Policy and the Roundtable on Genomics and Precision Health [45]. We highlighted seven key implementation challenges, which center on cost-effectiveness evidence; navigating patient and provider preferences and concerns; ensuring gender and ethnic diversity and representation in FH genetic testing; establishing consensus among clinical guidelines about FH genetic testing based on the latest and most relevant clinical research; and the need toestablish a clearer pathogenicity assessment of FH variants to more accurately assess CVD risk in FH populations with various phenotypes. Overcoming these implementation challenges is imperative to the mission of reducing CVD risk among the population of the United States.
